# Spraying Ethephon Effectively Increased Canopy Light Transmittance of Densely Planted Summer Maize, Thus Achieving Synergistic Improvement in Stalk Lodging Resistance and Grain Yield

**DOI:** 10.3390/plants11172219

**Published:** 2022-08-26

**Authors:** Wenjie Geng, Zhichao Sun, Baizhao Ren, Hao Ren, Bin Zhao, Peng Liu, Jiwang Zhang

**Affiliations:** State Key Laboratory of Crop Biology, College of Agronomy, Shandong Agricultural University, Tai’an 271018, China

**Keywords:** planting density, ethephon, summer maize, stalk lodging resistance, grain yield

## Abstract

Increasing planting density is an effective way to improve maize yield, but high plant populations often cause a lodging problem. This experiment was conducted to investigate the effect of increasing planting density on stalk lodging resistance and grain yield, and to explore the effects on stalk and yield properties of spraying ethephon in densely planted summer maize. The summer maize hybrid, Xundan20 (XD20), was used as experimental material. It was grown by spraying water (CK) or ethephon (E) at BBCH (BASF, Bayer, Ciba-Geigy and Hoechst) 17 under three different planting densities of 60,000 plants ha^−1^ (L), 75,000 plants ha^−1^ (M) and 90,000 plants ha^−1^ (H) in order to explore the possibility of synergistic improvement in stalk lodging resistance and grain yield. The results from this experiment suggested that the gravity center height of densely planted summer maize was significantly increased, the stem diameter, area and number of vascular bundles were significantly decreased and the dry weight per unit internode was significantly decreased, thereby weakening the stalk rind penetration strength and bending performance, resulting in a significant increase in lodging percentage. The ear height was significantly decreased and the SPAD (soil and plant analysis development) and canopy light transmittance were increased after spraying ethephon; then, the internode dry weight per unit length was increased and the stalk rind penetration strength and bending performance were enhanced so as to significantly reduce the lodging percentage and increase the grain yield. The correlation analysis further showed that lodging percentage was significantly negatively correlated with stem diameter, area and number of vascular bundles and stalk bending performance, but there were no strong relationships with grain yield. This suggested that the synergistic improvement in stalk lodging resistance and grain yield was not contradictory. Under the experiment conditions, the effect of spraying ethephon was most significant when the planting density was 90,000 plants ha^−1^. At the time, the lodging percentage and grain yield were 12.2% and 11,137.5 kg ha^−1^, which were decreased by 44.6% and increased by 8.0% compared with the control treatment. Scientific chemical regulation could significantly improve the stalk lodging resistance and grain yield of densely planted summer maize.

## 1. Introduction

Lodging is a common problem in maize production. It is mainly divided into root lodging and stalk lodging, which seriously affect the yield, quality and mechanized harvesting quality. During the grain filling stage of maize, the ear weight increases gradually, and the center of gravity increases accordingly [1]. At the same time, the stalk moisture gradually decreases, which reduces the expansion pressure of the cells, reduces the cell volume and increases the gap between the cells, resulting in a decrease in stalk strength [2]. Therefore, stalk lodging usually occurs during the grain filling stage, which destroys the crop canopy structure, weakens the leaves’ photosynthesis, affects the material transport between organs and results in final yield reductions of 45–48% [3,4,5]. Additionally, it reduces the accumulation of nutrients in grains, thus reducing the commodity quality [6]. Lodging also causes harvest obstacles and increases harvest costs [7], restricting the comprehensive utilization and industrialization development of maize. Therefore, more research is necessary to improve maize stalk lodging resistance.

Stem morphological characteristics, chemical components, anatomical structures and mechanical properties all affect the stalk strength, thus affecting the stalk lodging resistance. Usually, the more flexible stems have longer, thicker and conical internodes [8]. A high proportion of cell sclerenchyma was beneficial to plant lodging resistance [9]. Vascular bundles had a supporting effect on stems, but there were different understandings about the relationship between the number and area of vascular bundles and stalk strength in previous studies. Ren et al. [10] showed that the number of vascular bundles was significantly positively correlated with lodging resistance. Jiang et al. [11] showed that the increase in the number of vascular bundles was not conducive to lodging resistance, and the areas of vascular bundles and phloem were significantly positively correlated with stalk strength. Some people thought that hybrids with the area of smaller vascular bundles had stronger lodging resistance [12]. In previous studies, the content and composition of structural carbohydrates were important in lodging resistance, the accumulation of cellulose and lignin could increase stalk strength and lignin H subunits played a key role in strengthening the maize stalk [13]. Stalk bending strength was strongly associated with stalk lodging across multiple environments, and it was an excellent phenotype for predicting lodging [14].

Increasing planting density is an effective way to increase yield [15,16,17], but, once the planting density exceeds the optimal density, the maize yield will decrease [18]. In previous studies, high planting density increased leaf area index (LAI), effectively expanded group source and sink, improved group photosynthesis efficiency and increased crop productivity [19,20,21]. However, other studies showed that the ventilation and canopy light transmittance of the dense planting density maize group were reduced and individual competition was intensified, thereby accelerating leaf senescence and reducing dry matter accumulation [22,23,24,25]. At the same time, the plant height and gravity center height were increased, the stem was thin and weak and the root system was poorly developed, which would increase the probability of lodging and have a negative impact on the harvested yield [26,27].

Plant growth regulators play an important role in modulating diverse processes throughout plant growth and development. Ethephon and its compounding agents are widely used in densely planted maize to prevent lodging and increase yield [28]. Ethephon could regulate plant hormone biosynthesis and related signal transductions, thus inhibiting internode elongation, resulting in decreased plant height, ear height and gravity center height and increased dry weight per unit internode and increased stalk strength [29,30]. In previous studies, after ethephon application, stalk rind penetration strength, bending strength, lignin content and the key enzymes activities in lignin synthesis were significantly increased, which was beneficial to enhance stalk lodging resistance and increase the yield [31]. However, some studies showed that spraying ethephon in environments where lodging did not occur led to a reduction in maize yield [32,33].

Lodging resistance is an important condition for high yield, stable yield and mechanized production of densely planted maize. Increasing the planting density is an important way to increase the yield, but blindly increasing planting density increases the lodging risk and reduces the grain yield. Ethephon has been widely applied to reduce the lodging risk in maize production. However, there is little information on how ethephon regulates crop canopy structure and stalk dry matter accumulation to improve stalk strength. How to combine the two cultivation measures of increasing planting density and spraying ethephon to achieve the goal of synergistic improvement in yield and lodging resistance is the focus of future research. Compared with a previous study [34], we paid more attention to field agronomic traits, such as LAI, soil and plant analysis development (SPAD) and canopy light transmittance. Additionally, we used a new indicator named maximum bending strength in the field to reflect stalk strength. The focus of this study was to explore the possibility of achieving synergistic improvement in stalk lodging resistance and grain yield by spraying ethephon under dense planting density so as to provide a scientific basis for improving the efficiency and competitiveness of the maize industry.

## 2. Results

### 2.1. Effects of Spraying Ethephon on Plant Height, Ear Height and Gravity Center Height of Summer Maize

**Figure 1:**
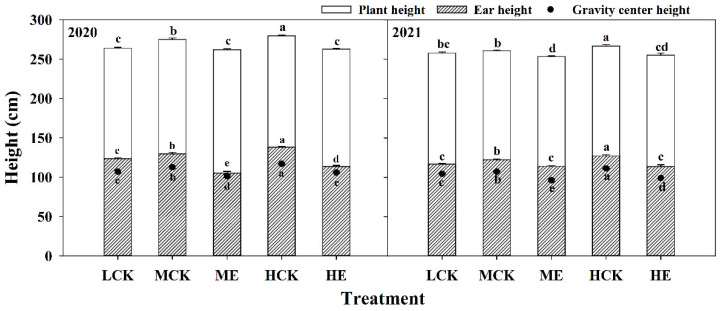
Effects of spraying ethephon on plant height, ear height and gravity center height of summer maize. LCK, MCK, ME, HCK and HE were the treatments of low density control, medium density control, medium density sprayed with ethephon, high density control and high density sprayed with ethephon, respectively. Different lowercase letters in the figure indicated significant difference at 5% level.

The plant height, ear height and gravity center height of Xundan20 (XD20) were significantly increased as planting density increased (Figure 1). Compared with those of low control density treatment (LCK), the plant height, ear height and gravity center height of medium control density treatment (MCK) and high density control treatment (HCK) were increased by 2.7%, 4.7%, 3.9%, 4.7%, 10.5% and 8.0%, respectively. The plant height, ear height and gravity center height of XD20 were significantly decreased after spraying ethephon (Figure 1). The plant height, ear height and gravity center height under ME were 3.7%, 12.9% and 10.1% lower than those under MCK, respectively; and those under HE were 5.2%, 14.3% and 10.2% lower than those under HCK, respectively. The results obtained from 2020 to 2021 exhibited similar basic trends.

### 2.2. Effects of Spraying Ethephon on Leaf Area Index (LAI) and Soil and Plant Analysis Development (SPAD) of Summer Maize

With the increase in planting density, the LAI of XD20 was significantly increased, and the SPAD was significantly decreased (Table 1). Compared with those of LCK, the LAI of MCK was increased by 26.7%, and the SPAD of MCK was decreased by 3.5%; the LAI of HCK was increased by 48.8%, and the SPAD of HCK was decreased by 6.7%. After spraying ethephon, the LAI of XD20 was significantly decreased, and the SPAD was significantly increased (Table 1). Compared with those of MCK, the LAI of medium density sprayed with ethephon treatment (ME) was decreased by 5.8%, and the SPAD of ME was increased by 2.3%; compared with those of HCK, the LAI of high density sprayed with ethephon treatment (HE) was decreased by 14.2%, and the SPAD of HE was increased by 3.7%. The results obtained from 2020 to 2021 exhibited similar basic trends.

### 2.3. Effects of Spraying Ethephon on Canopy Light Transmittance of Summer Maize

**Figure 2:**
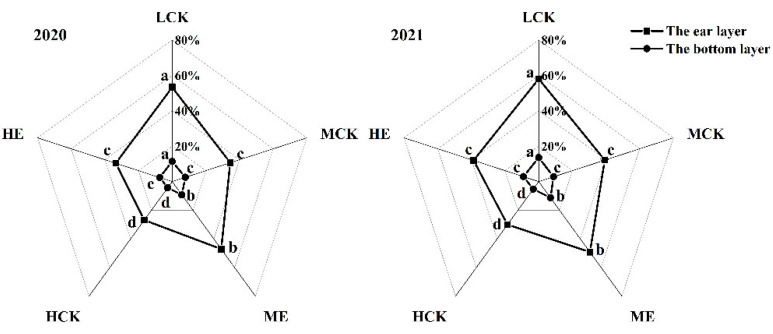
Effects of spraying ethephon on canopy light transmission of summer maize. LCK, MCK, ME, HCK and HE were the treatments of low density control, medium density control, medium density sprayed with ethephon, high density control and high density sprayed with ethephon, respectively. Different lowercase letters in the figure indicated significant difference at 5% level.

The canopy light transmittances of XD20 were significantly decreased of the ear layer and bottom layer as planting density increased (Figure 2). Compared with that of LCK, the canopy light transmittances of the ear layer and bottom layer of MCK and HCK were decreased by 33.8%, 33.3%, 48.9% and 61.7%, respectively. The canopy light transmittances of XD20 were significantly increased of the ear layer and bottom layer after spraying ethephon (Figure 2). The ear and bottom layers under ME were 31.1% and 23.9% higher than those under MCK, respectively; and those under HE were 26.8% and 72.9% higher than those under HCK, respectively. The results obtained from 2020 to 2021 exhibited similar basic trends.

### 2.4. Effects of Spraying Ethephon on Stalk Traits of Summer Maize

#### 2.4.1. Internode Length

The basal internode (the internode near the ground) length of XD20 was significantly increased as planting density increased (Table 2). Compared with that of LCK, the basal third internode (the third internode counted from bottom to top above the ground) lengths of MCK and HCK were increased by 13.0% and 24.8%, respectively. The basal internode length of XD20 was significantly decreased after spraying ethephon (Table 2). The basal third internode lengths under ME and HE were 14.0% and 20.2% lower than those under MCK and HCK, respectively. The results obtained from 2020 to 2021 showed that the basal internode lengths exhibited similar basic trends.

#### 2.4.2. Stem Diameter

The basal stem diameter of XD20 was significantly decreased as planting density increased (Table 3). Compared with that of LCK, the basal third stem diameters of MCK and HCK were increased by 4.4% and 10.7%, respectively. The basal stem diameter of XD20 was significantly increased after spraying ethephon (Table 3). The basal third stem diameters under ME and HE were 5.3% and 11.0% higher than those under MCK and HCK, respectively. The results obtained from 2020 to 2021 showed that the basal internode lengths exhibited similar basic trends.

#### 2.4.3. Internode Dry Weight per Unit Length

**Figure 3:**
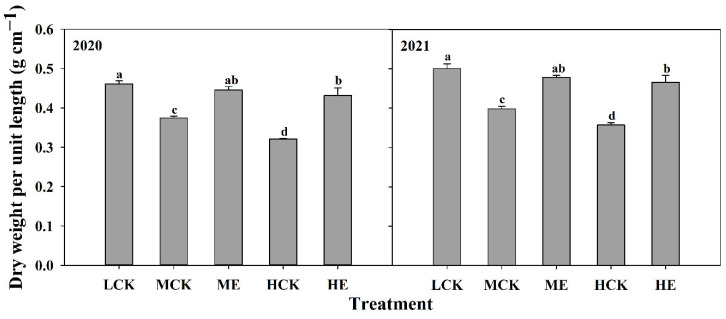
Effects of spraying ethephon on dry weight per unit length of the basal third internode. LCK, MCK, ME, HCK and HE were the treatments of low density control, medium density control, medium density sprayed with ethephon, high density control and high density sprayed with ethephon, respectively. Different lowercase letters in the figure indicated significant difference at 5% level.

The basal third internode dry weight per unit length of XD20 was significantly decreased as planting density increased (Figure 3). Compared with that of LCK, the basal third internode dry weights per unit length of MCK and HCK were decreased by 19.7% and 29.5%, respectively. The basal third internode dry weight per unit length of XD20 significantly increased after spraying ethephon (Figure 3). The basal third internode dry weights per unit length under ME and HE were 19.6% and 32.3% higher than those under MCK and HCK, respectively. The results obtained from 2020 to 2021 exhibited similar basic trends.

#### 2.4.4. Stem Anatomical Structure

**Figure 4:**
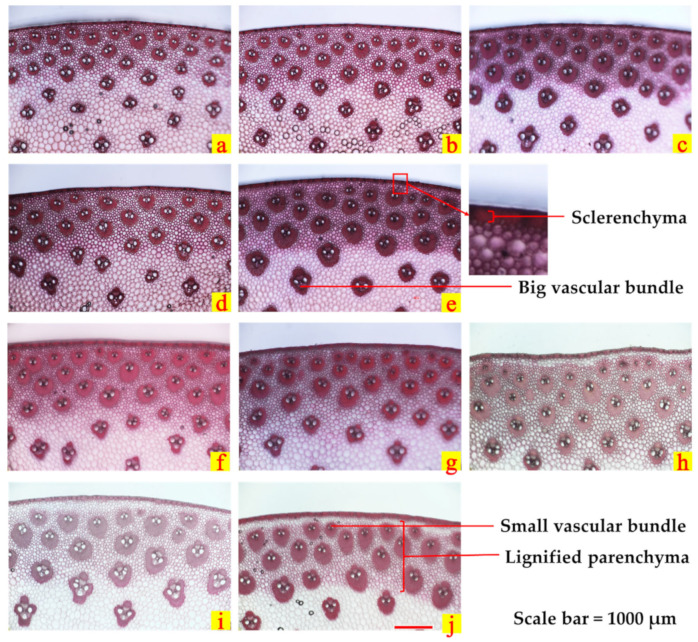
Effects of spraying ethephon on anatomical structure of the basal third internode. (**a**–**e**), respectively, showed the microstructure at LCK, MCK, ME, HCK and HE in 2020; (**f**–**j**), respectively, showed the microstructure at LCK, MCK, ME, HCK and HE in 2021. Scale bar = 1000 μm.

With the increase in planting density, the thickness of sclerenchyma and lignified parenchyma and the area and number of vascular bundles of XD20 were significantly decreased (Figure 4, Table 4). Compared with those of LCK, the thickness of sclerenchyma, the thickness of lignified parenchyma, the area of small vascular bundles, the area of large vascular bundles, the number of small vascular bundles and the number of large vascular bundles of MCK were decreased by 13.7%, 8.6%, 8.5%, 4.9%, 14.0% and 19.9%, respectively. Additionally, those of HCK were decreased by 30.5%, 20.1%, 20.1%, 19.5%, 24.8% and 37.4%, respectively. After spraying ethephon, the thickness of sclerenchyma and lignified parenchyma of XD20 were significantly increased, the number of vascular bundles was significantly increased and the area of small vascular bundles was significantly increased. The thickness of sclerenchyma, the thickness of lignified parenchyma, the area of small vascular bundles, the area of large vascular bundles and the number of small vascular bundles under ME were 16.5%, 5.4%, 9.4%, 5.1% and 6.3% higher than those under MCK, respectively; and those under HE were 43.4%, 19.0%, 18.2%, 24.3% and 23.5% higher than those under HCK, respectively. Compared with the equal density control treatment, the number of large vascular bundles was significantly increased in 2020, but there was no significant difference in 2021. The experimental results of the two years were different.

#### 2.4.5. Stalk Rind Penetration Strength

**Figure 5:**
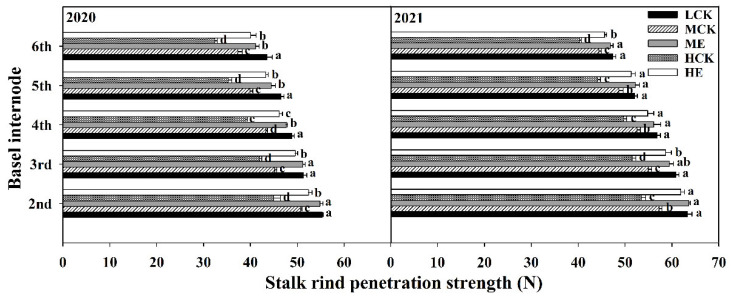
Effects of spraying ethephon on stalk rind penetration strength of summer maize. LCK, MCK, ME, HCK and HE were the treatments of low density control, medium density control, medium density sprayed with ethephon, high density control and high density sprayed with ethephon, respectively. Different lowercase letters in the figure indicated significant difference at 5% level.

The stalk rind penetration strength of XD20 significantly decreased as planting density increased (Figure 5). Compared with that of LCK, the basal third internode rind penetration strengths of MCK and HCK were decreased by 10.8% and 16.7%, respectively. The stalk rind penetration strength of XD20 was significantly increased after spraying ethephon (Figure 5). The basal third internode rind penetration strengths under ME and HE were 10.7% and 16.0% higher than those under MCK and HCK, respectively. The results obtained from 2020 to 2021 showed that the basal internode lengths exhibited similar basic trends.

#### 2.4.6. Maximum Bending Strength in the Field

**Figure 6:**
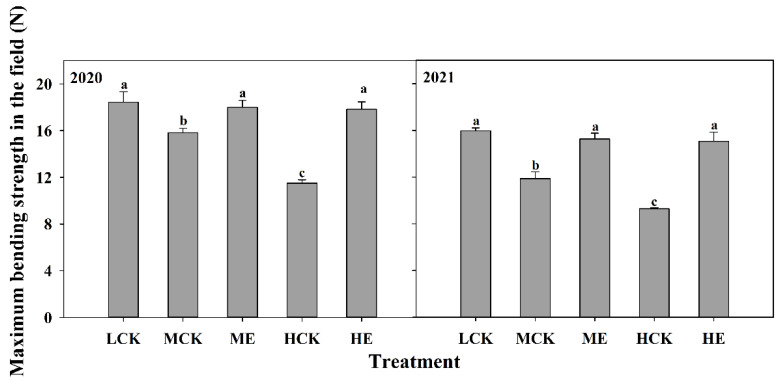
Effects of spraying ethephon on maximum bending strength in the field of summer maize. LCK, MCK, ME, HCK and HE were the treatments of low density control, medium density control, medium density sprayed with ethephon, high density control and high density sprayed with ethephon, respectively. Different lowercase letters in the figure indicated significant difference at 5% level.

The maximum bending strength in the field of XD20 was significantly decreased as planting density increased (Figure 6). Compared with that of LCK, the maximum bending strengths in the field of MCK and HCK were decreased by 19.9% and 39.7%, respectively. The maximum bending strength in the field of XD20 was significantly increased after spraying ethephon (Figure 6). The maximum bending strengths in the field under ME and HE were 21.2% and 58.5% higher than those under MCK and HCK, respectively. The results obtained from 2020 to 2021 exhibited similar basic trends.

### 2.5. Effects of Spraying Ethephon on Lodging Percentage of Summer Maize

**Figure 7:**
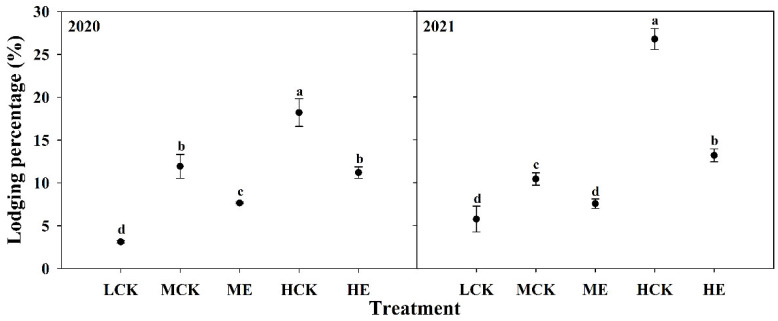
Effects of spraying ethephon on lodging percentage of summer maize. LCK, MCK, ME, HCK and HE were the treatments of low density control, medium density control, medium density sprayed with ethephon, high density control and high density sprayed with ethephon, respectively. Different lowercase letters in the figure indicated significant difference at 5% level.

The lodging percentage of XD20 was significantly increased as planting density increased (Figure 7). Compared with that of LCK, the lodging percentages of MCK and HCK were increased by 181.4% and 481.7%, respectively. The lodging percentage of XD20 was significantly decreased after spraying ethephon (Figure 7). The lodging percentages under ME and HE were 31.8% and 44. 6% lower than those under MCK and HCK, respectively. The results obtained from 2020 to 2021 exhibited similar basic trends.

### 2.6. Effects of Spraying Ethephon on Grain Yield of Summer Maize

Compared with that of LCK, the grain yields of MCK and HCK were significantly increased in XD20, and there was no significant difference in grain yield between MCK and HCK (Table 5). With the increase in planting density, the ears per hectare, the kernels per ear and 1000-grain weight of XD20 all showed a downward trend. After spraying ethephon, the grain yields of ME and HE of XD20 were significantly increased compared with the equal density control treatment, which were 3.6% and 8.0%, respectively (Table 5). After spraying ethephon, the ears per hectare and 1000-grain weight in XD20 were increased and decreased, respectively, and the kernels per ear showed different changes in two years.

### 2.7. Correlation Analysis

**Figure 8:**
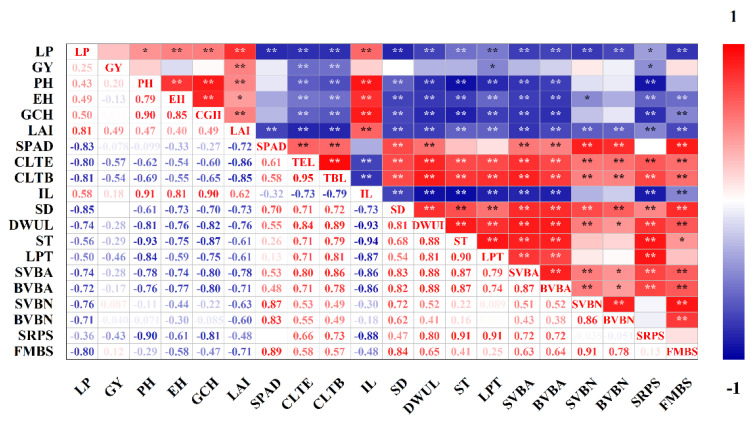
Correlation analysis. LP, lodging percentage; GY, grain yield; PH, plant height; EH, ear height; GCH, gravity center height; CLTE, canopy light transmittance of the ear layer; CLTB, canopy light transmittance of the bottom layer; IL, internode length; SD, stalk diameter; DWUL, dry weight per unit length; ST, sclerenchyma thickness; LPT, lignified parenchyma thickness; SVBA, the area of small vascular bundles; BVBA, the area of big vascular bundles; SVBN, the number of small vascular bundles; BVBN, the number of big vascular bundles; SRPS, stalk rind penetration strength; FMBS, maximum bending strength in the field. *, significant at 0.05 probability level. **, significant at 0.01 probability level.

Correlation analysis was carried out between the data of the basal third internode and other measured indicators (Figure 8). The results showed that the lodging percentage was significantly negatively correlated with the canopy light transmittances of the ear layer and the bottom layer, and the correlation coefficients were −0.80 and −0.81, respectively. LAI, SPAD and internode dry weight per unit length were also extremely significantly correlated with lodging percentage, with correlation coefficients of 0.81, −0.83 and −0.74, respectively. The characteristics of vascular bundles were significantly negatively correlated with the lodging percentage. The correlation coefficients of the area of small vascular bundles, the area of large vascular bundles, the number of small vascular bundles, the number of large vascular bundles and the lodging percentage were −0.74, −0.72, −0.76 and −0.71, respectively. Stem diameter and maximum bending strength in the field also significantly affected stalk lodging resistance, and the correlation coefficients with lodging percentage were −0.85 and −0.80. Grain yield was significantly positively correlated with LAI and significantly negatively correlated with canopy light transmittance.

## 3. Discussion

### 3.1. Increasing Planting Density Significantly Reduced Stalk Lodging Resistance of Summer Maize

With the increase in planting density, the LAI increased and the plants shaded each other, which significantly reduced the photosynthetically active radiation to the ear layer and the bottom layer. The light spectral distribution within the canopy becomes depleted of wavelengths between 400 and 700 nm, which are absorbed by chlorophyll, whereas flux density in the far-red (>700 nm) remains high. The red:far-red ratio is decreased, which induces the shade avoidance response [35,36], thus promoting internode elongation, reducing stem diameter and reducing the accumulation of carbohydrates in basal internodes, resulting in a decrease in internode dry weight per unit length, significantly reducing stalk mechanical strength. The results of stem anatomical structure showed that increasing planting density decreased the sclerenchyma and lignified parenchyma thickness and reduced the area and number of vascular bundles, thereby weakening the stalk mechanical strength. Those changes would also lead to a decrease in stem flow, which negatively affected grain yield [37]. Stalk rind penetration strength was significantly correlated with stalk lodging resistance [38]. Additionally, stalk bending strength was determined by the cumulative effect of stem metabolic and morphological properties, which could predict 81% of the variation in stalk strength [14,39]. The results of our experiment showed that increasing the planting density significantly reduced the stalk rind penetration strength and field maximum bending performance and increased the lodging percentage. Taken together, increasing the planting density significantly decreased the stalk lodging resistance of summer maize, thus increasing the lodging percentage.

### 3.2. The Regulation of Spraying Ethephon on Stalk Lodging Resistance and Grain Yield of Densely Planted Summer Maize

Spraying ethephon reduced LAI, improved canopy light transmittance and increased leaf SPAD, thereby enhancing leaf photosynthetic capacity, conducive to stem dry matter accumulation and stalk strength formation. Ethephon could promote the accumulation of ethylene in stems, reduce the concentrations of auxin and gibberellin and increase the expression of secondary cell wall genes [30]. As a result, the internode length was reduced, the stem diameter was increased and the stalk strength was improved. The correlation analysis showed that stalk rind penetration strength was significantly positively correlated with sclerenchyma thickness and lignified parenchyma thickness. Additionally, stem diameter, vascular bundle characteristics and maximum bending strength in the field were significantly negatively correlated with lodging percentage. After spraying ethephon, the sclerenchyma and lignified parenchyma thickness of the stem was thickened, thereby significantly improving the rind penetration strength and bending strength. The number of large vascular bundles showed differences within two years, which may be caused by the wide genetic variation in vascular bundle characteristics [40]. In a previous study, the effect of the number of vascular bundles on lodging was related to the elastic modulus of vascular bundles [41], and the effect of vascular bundle system on stalk strength should be compared and analyzed according to specific conditions [4]. The results of this experiment showed that the lodging percentages of spraying ethephon under the two densities were significantly reduced, and the maize stalk lodging resistances were significantly enhanced.

Yield is affected by various factors. The ideal planting density optimizes the relationship between the number of ears per hectare, kernels per ear and 1000-grain weight so as to play to the group advantage and improve the yield potential. The results of the current experiment showed that canopy light transmittance was significantly negatively correlated with grain yield across a range of planting densities (Table 5). However, under the condition of high density, the decrease in canopy light transmittance does not mean the full utilization of light, and the mutual shading of plants wastes more light resources. Spraying ethephon increased canopy light transmittance and significantly increased light energy utilization, thereby increasing the dry matter accumulation of the population and achieving a yield increase. Therefore, the key to high grain yield was to make full use of light energy. The grain yield was not significantly increased from 75,000 plants ha^−^^1^ to 90,000 plants ha^−^^1^, which may be related to plant-to-plant competition and the increase in lodging percentage. In a previous study, ethephon affected the grain filling characteristics, decreased the grain storage capacity and activity and thus reduced the grain yield [42]. Compared with the equal density control treatment, the grain yield increase with spraying ethephon treatment under 75,000 plants ha^−^^1^ was significantly lower than that under 90,000 plants ha^−^^1^. which might be related to the increase in ears per hectare under 75,000 plants ha^−^^1^ not being able to significantly compensate for the negative effect of ethephon on ear development. In this experiment, we found some differences in spike traits, such as spike length, among treatments, but there was no significant difference in days to male flowering and days to female flowering under field conditions among the different treatments, which was consistent with other previous studies [43,44,45]. The different trend of kernels per ear in two years may be caused by the higher ears per hectare in 2021 and the adaptive change in yield components. The results of our experiment showed that spraying ethephon significantly (*p* < 0.05) increased the number of ears per hectare in both years at both 75,000 plants ha^−^^1^ and 90,000 plants ha^−^^1^, and it significantly (*p* < 0.05) increased the grain yield at both 75,000 plants ha^−^^1^ and 90,000 plants ha^−^^1^ in 2020, and at 90,000 plants ha^−^^1^ in 2021.

### 3.3. The Possibility of Synergistic Improvement in Stalk Lodging Resistance and Grain Yield of Densely Planted Summer Maize

In a previous study, researchers suggest that maize stalk structure requires more tissue to support its own mass and external loads when the crop is subjected to wind and rain, which reduces the potential biomass available for grain filling, reduces the harvest index and increases the risk of lodging [46]. Yield loss due to stalk lodging can be up to 75% [47,48]. For every 1% increase in lodging percentage, the ear drop percentage increased by 0.15%, significantly reducing the quality and speed of mechanized harvesting [49]. Therefore, it is necessary to coordinately improve the stalk lodging resistance and grain yield of densely planted maize. 

Effective utilization of solar radiation is the key method to coordinate stalk lodging resistance and grain yield. Solar radiation was the basis of plant growth and development, and 90% of the yield came from assimilates after the silking period [50]. In previous studies, insufficient light during the grain filling stage would not only reduce photosynthesis and affect grain development but also increased the transport of carbohydrates in the stem, further reducing stalk strength [51]. After spraying ethephon, the internode length was decreased, which was also thought to reduce the distance between source and sink, weaken the storage capacity of the stem and alleviate the competition between stalk growth and ear development, thus contributing to yield increase [32,52]. Taken together, adequate light penetration into the crop canopy could prolong the leaf functional period, promote the formation of stem strength in the early stage and slow down the senescence rate of plants in the later stage. It also reduced the remobilization of the outward transport of material storage in the stem so as to optimize the distribution of assimilates in the ear and stem, synergistically improving stalk lodging resistance and grain yield [53,54]. 

Scientific chemical regulation is an important strategy to achieve synergistic improvement in stalk lodging resistance and grain yield of densely planted maize. In other studies, delaying application of ethephon avoided negative effects on floret initiation and development of florets in the early stage of vegetative growth, and it improved yield by decreasing stem competition with ears for assimilates, but the lodging prevention effect would decrease [32]. Huang et al. [55] showed that application of DHEAP (N,N-Diethyl-2-hexanoyl oxygen radicals-ethyl amine (2-ethyl chloride) phosphonic acid salt) constructed compact plant type, increased the average leaf inclination of leaves above ear position, improved the net photosynthetic rate and key photosynthetic enzyme activities of ear leaves and achieved high and stable yield. The results of this experiment showed that LAI and light transmittance were significantly correlated with lodging percentage and grain yield. Spraying ethephon decreased the LAI and increased the SPAD, which was beneficial for the efficient use of solar radiation. Additionally, there was no significant negative correlation between stalk lodging resistance and grain yield, so the synergistic improvement in them was not contradictory. Planting density and spraying ethephon have obvious interaction effects on various factors, and we can explore a reasonable combination of density and ethephon to achieve the synergistic improvement in stalk lodging resistance and grain yield. 

Other previous studies have shown that the effects of increasing density and spraying ethephon on different maize hybrids were extensive and basically consistent [31,56,57,58]. We used Xundan20, Denghai605 and Denghai618 in our previous study, and the regulations of increasing density and spraying ethephon on stalk lodging resistance were consistent. Increasing planting density and spraying ethephon had more obvious effects on Xundan20. Therefore, we selected Xundan20 to explore how to synergistically improve stalk lodging resistance and grain yield. 

## 4. Materials and Methods

### 4.1. Materials

Xundan20 (XD20) is a medium maturing summer maize hybrid with the suitable planting density of 60,000–67,500 plants ha^−^^1^ [59]. It has the characteristics of good fecundity, fast filling and excellent quality [60]. It used to be the second maize hybrid on planting area in China, but its lodging resistance and disease resistance decreased significantly with the increase in planting density [59,60].

### 4.2. Experimental Design

**Figure 9:**
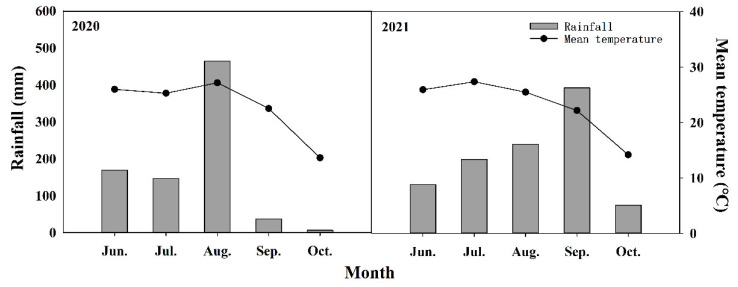
Rainfall and mean temperature during the experimental period.

The experiment was conducted at the experimental farm of Shandong Agricultural University (17.16° E, 36.16° N) from 2020 to 2021 using XD20 as experimental material. The region climate was a temperate monsoon climate, and Figure 9 shows the rainfall and mean temperature during the experimental period. The rainfall and mean temperature during the experimental period were 823.3 mm and 22.8 °C in 2020, and 1402.7 mm and 23.0 °C in 2021. The soil at the experimental site was classified as brown loam, and the contents of organic matter, total nitrogen (N), available phosphorus (P) and available potassium kalium (K) in the 0–20 cm soil layer were 12.01 g kg^−^^1^, 0.74 g kg^−^^1^, 41.06 mg kg^−^^1^ and 103.89 mg kg^−^^1^. The experiment was arranged as a randomized complete block design. There were 3 plots per treatment, the plots were 3 m × 15 m and there were 5 rows in a plot. A row spacing of 60 cm was used for all planting densities, while plant spacings within rows were set at 27.8, 22.2 and 18.5 cm for 60,000, 75,000 and 90,000 plants ha^−^^1^, respectively. The densities were set at three gradients of 60,000 plants ha^−^^1^, 75,000 plants ha^−^^1^ and 90,000 plants ha^−^^1^. Ethephon (Anyang Quanfeng Biological Technology Co., Ltd., Anyang, China; 40% active ingredient, used 180 mL per hectare and diluted 1500 times with water) was sprayed at BBCH (BASF, Bayer, Ciba-Geigy and Hoechst) 17; the control (CK) was sprayed with water. A total of 300 kg ha^−^^1^ N (urea, 46% N), 120 kg ha^−^^1^ P (superphosphate, 17% P_2_O_5_) and 240 kg ha^−^^1^ K (potassium chloride, 60% K_2_O) were applied, aiming at the yield level of 12,000 kg ha^−1^. Nitrogen fertilizer was split into two applications, 40% at BBCH 16 and 60% at BBCH 37. Total P and K doses were applied in a single application at BBCH 16. The experiment was under the local agricultural management in order to ensure efficient water and to avoid weeds, pests or diseases.

The plot area was 45 m^2^ and includes 207–405 plants, and we divided sampling areas in each plot and marked sampling plants at BBCH 16. Therefore, our experiment ensures consistency in density.

### 4.3. Measurement Items and Methods

#### 4.3.1. Plant Height, Ear Height and Gravity Center Height

At the milking stage (R3), 6 representative plants were selected from each plot. The lengths from the base to the highest position of the plant and the node bearing the ear of the plant were considered the plant height and ear height. The gravity center height was examined by placing the maize across an outstretched fulcrum and moving the stalk along the fulcrum until the balance point was reached, and the distance between the base and the center of gravity was gravity center height. Measurements were performed manually with a tape.

#### 4.3.2. LAI and SPAD

At the tasseling stage (VT), 6 representative plants were selected from each plot. The leaf length and maximum leaf width of each leaf were measured with a tape, and leaf area index was calculated according to the formula. A portable chlorophyll meter (SPAD-502, Minolta Camera Co., Ltd., Osaka, Japan) was used to determine the SPAD of ear leaf.

Single leaf area (cm^2^) = leaf length × leaf width × 0.75

Leaf area index = (leaf area per plant × number of plants)/plot area

#### 4.3.3. Crop Canopy

At the VT stage, a canopy analysis system (Sunscan, Delta, UK) was used to measure the light radiation characteristics within the canopy between rows. Photosynthetically active radiation of ear layer (ear position) and bottom layer (10 cm above the ground) were collected, respectively, to calculate the light transmittance.

Light transmittance (%) = photosynthetically active radiation at ear level or bottom layer/total solar radiation intensity × 100

#### 4.3.4. Internode Length and Stem Diameter

At the R3 stage, 6 representative plants were selected in each plot. The internode lengths of the basal second, third, fourth, fifth and sixth internodes were measured with a tape. The stem diameters of the basal second, third, fourth, fifth and sixth internodes were measured with a digital Vernier caliper.

#### 4.3.5. Internode Dry Weight per Unit Length

At the R3 stage, 3 representative plants were selected from each plot. The basal third internode was placed into a paper bag, and the internodes were subjected to enzyme deactivation at 105 °C for 30 min and then dried at 80 °C to a constant weight, which was recorded as the dry weight. The internode dry weight per unit length (cm) was calculated according to the basal third internode dry weight and internode length.

Internode dry weight per unit length (g cm^−1^) = internode dry weight/internode length

#### 4.3.6. Stem Anatomical Structure

At the R3 stage, 3 representative plants were selected in each plot, and the middle 2 cm portion of the basal third internode was fixed with Carnot and stored in 70% ethanol. Hand-slicing and saffron staining were used to observe and photograph the structure of vascular bundles in the stem using a fluorescence microscope camera system (Ni-u, Nikon, Japan). Sclerenchyma thickness, lignified parenchyma thickness, the number of vascular bundles and the area of vascular bundles were measured using the measurement function of that camera system. The number of vascular bundles per plant were calculated per unit area. The number of vascular bundles on the 1/8 area of stem cross section was counted, and then the total number of vascular bundles was calculated.

#### 4.3.7. Stalk Rind Penetration Strength

At the R3 stage, 6 representative plants were selected in each plot. The rind penetration strengths of the basal second, third, fourth, fifth and sixth internodes were determined with a stalk strength tester (YYD-1, Zhejiang Top Cloud-Agri Technology Co., Ltd., Zhenjiang, China). For these measurements, the internodes were horizontally positioned on the supportive columns of the stalk strength tester, and the instrument probe was vertically applied at the mid internode. Stalk rind penetration strength was expressed in Newtons (N).

#### 4.3.8. Maximum Bending Strength in the Field

At the R3 stage, 6 representative plants were selected in each plot. The field portable lodging resistance tester (Laizhou Kaitian Instrument Co., Ltd., Laizhou, China) was fixed at the position of ear, and the instrument was pulled to make the plant bend to one side. The instrument automatically recorded the force on the plant when the stalk broke.

#### 4.3.9. Lodging Percentage

The lodging percentage of each plot was investigated after maize lodging, and the ratio of lodging (angle between stalk and ground < 30° or stalk broken) of each plot to the total number of plants in the plot was the lodging percentage.

Lodging percentage (%) = number of lodging plants/total number of plants in the plot × 100

#### 4.3.10. Grain Yield

The number of ears per hectare was determined using field trait surveys, and 30 ears from the middle three rows of plants in each plot were harvested to determine the numbers of grain rows per ear and kernel per row. The yield was calculated as follows.

Grain yield (kg ha^−1^) = ears (ears ha^−1^) × kernels per ear × grain weight (g) × 10^−6^/(1 − 14%)

where grain weight was calculated as the average grain weight based on the 1000-grain weight.

#### 4.3.11. Data Analysis

Microsoft Excel 2019 and SPSS 26 software were used for statistics and analysis, and SigmaPlot 14.0 and Origin 2021 software were used for graphing. One-way ANOVA and Duncan’s method were used for analysis of variance and multiple comparisons (α = 0.05), and Pearson’s method was used for correlation analysis of each index.

## 5. Conclusions

The stalk lodging resistance of XD20 was significantly decreased as planting density increased. The canopy light transmittance, the quality of basal internodes and the lodging resistance of the stem were improved after spraying ethephon. Under the experiment conditions, the effect of spraying ethephon was the most significant when the planting density was 90,000 plants ha^−1^. At the time, the lodging percentage and grain yield were 12.2% and 11,137.5 kg ha^−1^, which were decreased by 44.6% and increased by 8.0% compared with the control treatment. Scientific chemical regulation can improve the lodging resistance of densely planted summer maize and achieve a high and stable yield.

## Figures and Tables

**Table 1:** Effects of spraying ethephon on LAI and SPAD of summer maize.

Treatment	LAI		SPAD	
	2020	2021	2020	2021
LCK	4.2 ^d^	4.4 ^d^	60.4 ^a^	58.6 ^a^
MCK	5.3 ^c^	5.7 ^b^	58.9 ^b^	56.0 ^b^
ME	5.3 ^c^	5.0 ^cd^	59.1 ^b^	58.4 ^a^
HCK	6.4 ^a^	6.4 ^a^	56.5 ^c^	54.6 ^c^
HE	5.7 ^b^	5.3 ^bc^	58.6 ^b^	56.5 ^b^
ANOVA				
Year (Y)	ns		**	
Density (D)	**		**	
Ethephon (E)	**		**	
Y × D	ns		ns	
Y × E	*		*	
D × E	*		ns	
Y × D × E	ns		**	

LCK, MCK, ME, HCK and HE were the treatments of low density control, medium density control, medium density sprayed with ethephon, high density control and high density sprayed with ethephon, respectively. Different lowercase letters in the table indicated significant difference at 5% level. ns, no significance. *, significant at 0.05 probability level. **, significant at 0.01 probability level.

**Table 2:** Effects of spraying ethephon on internode length (cm) of summer maize.

Year	Treatment	2nd	3rd	4th	5th	6th
2020	LCK	7.5 ^bc^	10.3 ^c^	13.4 ^c^	15.5 ^d^	17.4 ^cd^
	MCK	8.2 ^b^	11.6 ^b^	14.2 ^b^	16.7 ^b^	18.4 ^b^
	ME	6.9 ^c^	10.0 ^c^	12.9 ^c^	15.5 ^d^	16.7 ^d^
	HCK	9.4 ^a^	12.8 ^a^	15.4 ^a^	18.0 ^a^	19.5 ^a^
	HE	7.8 ^b^	10.4 ^c^	13.0 ^c^	16.0 ^c^	17.7 ^bc^
2021	LCK	5.9 ^b^	9.2 ^c^	12.0 ^c^	14.0 ^c^	15.4 ^cd^
	MCK	6.3 ^b^	10.4 ^b^	13.5 ^b^	15.3 ^b^	16.5 ^b^
	ME	5.8 ^b^	8.9 ^c^	11.9 ^c^	13.8 ^c^	14.9 ^d^
	HCK	7.4 ^a^	11.5 ^a^	14.5 ^a^	16.1 ^a^	17.9 ^a^
	HE	6.2 ^b^	9.0 ^c^	12.0 ^c^	13.9 ^c^	15.7 ^b^
ANOVA						
Year (Y)		**	**	**	**	**
Density (D)		**	**	**	**	**
Ethephon (E)		**	**	**	**	**
Y × D		ns	ns	ns	ns	ns
Y × E		ns	ns	ns	ns	ns
D × E		ns	**	**	**	ns
Y × D × E		ns	ns	ns	ns	ns

LCK, MCK, ME, HCK and HE were the treatments of low density control, medium density control, medium density sprayed with ethephon, high density control and high density sprayed with ethephon, respectively. Different lowercase letters in the table indicated significant difference at 5% level. ns, no significance. **, significant at 0.01 probability level.

**Table 3:** Effects of spraying ethephon on stem diameter (mm) of summer maize.

Year	Treatment	2nd	3rd	4th	5th	6th
2020	LCK	24.8 ^a^	23.3 ^a^	22.1 ^a^	20.9 ^a^	20.2 ^a^
	MCK	23.1 ^c^	22.0 ^b^	20.9 ^b^	20.3 ^a^	19.3 ^b^
	ME	24.8 ^a^	23.4 ^a^	22.1 ^a^	20.8 ^a^	20.3 ^a^
	HCK	22.1 ^d^	21.0 ^c^	19.9 ^c^	19.3 ^b^	18.5 ^c^
	HE	24.0 ^b^	23.1 ^a^	22.1 ^a^	20.7 ^a^	19.7 ^ab^
2021	LCK	25.0 ^a^	23.0 ^ab^	22.1 ^a^	21.4 ^a^	20.4 ^a^
	MCK	23.7 ^bc^	22.3 ^b^	21.1 ^b^	20.4 ^c^	19.6 ^b^
	ME	24.8 ^ab^	23.2 ^a^	22.2 ^a^	21.2 ^a^	20.7 ^a^
	HCK	22.8 ^c^	20.4 ^c^	19.8 ^c^	19.6 ^d^	18.5 ^c^
	HE	24.3 ^ab^	22.8 ^ab^	21.9 ^ab^	20.9 ^b^	20.1 ^ab^
ANOVA						
Year (Y)		ns	ns	ns	ns	ns
Density (D)		**	**	**	**	**
Ethephon (E)		**	**	**	**	**
Y × D		ns	ns	ns	ns	ns
Y × E		ns	ns	ns	ns	ns
D × E		ns	**	*	*	ns
Y × D × E		ns	ns	ns	ns	ns

LCK, MCK, ME, HCK and HE were the treatments of low density control, medium density control, medium density sprayed with ethephon, high density control and high density sprayed with ethephon, respectively. Different lowercase letters in the table indicated significant difference at 5% level. ns, no significance. *, significant at 0.05 probability level. **, significant at 0.01 probability level.

**Table 4:** Effects of spraying ethephon on anatomical structure of the basal third internode.

Year	Treatment	Tissue Thickness		The Area of Vascular Bundles (mm^2^)		The Number of Vascular Bundles (No.)	
		Sclerenchyma (μm)	Lignified Parenchyma (mm)	Small Vascular Bundles	Big Vascular Bundles	Small Vascular Bundles	Big Vascular Bundles
2020	LCK	52.7 ^a^	1.67 ^a^	0.17 ^a^	0.20 ^a^	426 ^a^	199 ^a^
	MCK	45.3 ^b^	1.56 ^a^	0.16 ^b^	0.19 ^b^	365 ^c^	156 ^c^
	ME	52.6 ^a^	1.63 ^a^	0.17 ^a^	0.20 ^a^	404 ^b^	177 ^b^
	HCK	34.2 ^c^	1.36 ^b^	0.13 ^c^	0.16 ^c^	318 ^d^	123 ^d^
	HE	52.4 ^a^	1.57 ^a^	0.16 ^b^	0.20 ^ab^	401 ^b^	185 ^ab^
2021	LCK	62.0 ^a^	1.98 ^a^	0.18 ^a^	0.21 ^a^	374 ^a^	175 ^a^
	MCK	53.7 ^b^	1.77 ^b^	0.16 ^b^	0.20 ^a^	323 ^c^	143 ^b^
	ME	62.8 ^a^	1.88 ^a^	0.18 ^a^	0.21 ^a^	329 ^c^	137 ^b^
	HCK	45.9 ^c^	1.55 ^c^	0.15 ^c^	0.17 ^b^	283 ^d^	111 ^c^
	HE	61.3 ^a^	1.90 ^a^	0.17 ^a^	0.21 ^a^	342 ^b^	106 ^c^
ANOVA							
Year (Y)		**	**	**	**	**	**
Density (D)		**	**	**	**	**	**
Ethephon (E)		**	**	**	**	**	**
Y × D		ns	ns	*	ns	ns	**
Y × E		ns	ns	ns	ns	**	**
D × E		**	**	**	**	**	ns
Y × D × E		ns	ns	ns	ns	ns	**

LCK, MCK, ME, HCK and HE were the treatments of low density control, medium density control, medium density sprayed with ethephon, high density control and high density sprayed with ethephon, respectively. Different lowercase letters in the table indicated significant difference at 5% level. ns, no significance. *, significant at 0.05 probability level. **, significant at 0.01 probability level.

**Table 5:** Effects of spraying ethephon on grain yield and yield components of summer maize.

Year	Treatment	Ears (No. ha^−1^)	Kernels per Ear	1000-Grain Weight (g)	Grain Yield (kg ha^−1^)
2020	LCK	50,556 ^d^	598 ^a^	315.4 ^a^	9535.3 ^d^
	MCK	60,556 ^c^	545 ^c^	306.1 ^b^	10,102.2 ^cd^
	ME	66,667 ^b^	554 ^b^	291.1 ^c^	10,751.3 ^b^
	HCK	69,444 ^b^	526 ^e^	291.8 ^c^	10,658.7 ^bc^
	HE	75,556 ^a^	538 ^d^	289.1 ^c^	11,751.7 ^a^
2021	LCK	56,667 ^e^	601 ^a^	253.5 ^a^	8633.4 ^c^
	MCK	70,741 ^d^	572 ^b^	245.9 ^b^	9950.1 ^b^
	ME	73,704 ^c^	573 ^b^	237.2 ^c^	10,017.5 ^ab^
	HCK	77,593 ^b^	546 ^c^	234.8 ^d^	9947.5 ^b^
	HE	88,148 ^a^	528 ^c^	226.1 ^e^	10,523.2 ^a^
ANOVA					
Year (Y)		**	**	**	**
Density (D)		**	**	**	**
Ethephon (E)		**	ns	**	**
Y × D		ns	**	*	ns
Y × E		ns	*	ns	*
D × E		*	ns	**	ns
Y × D × E		*	ns	**	ns

LCK, MCK, ME, HCK and HE were the treatments of low density control, medium density control, medium density sprayed with ethephon, high density control and high density sprayed with ethephon, respectively. Different lowercase letters in the table indicated significant difference at 5% level. ns, no significance. *, significant at 0.05 probability level. **, significant at 0.01 probability level.

## Data Availability

All data generated or analyzed during this study are included in this published article.
